# Case Report: Repeated low doses of psilocybin reduce perceived symptom severity but fail to restore cognitive flexibility in a case of severe obsessive-compulsive disorder: an observational case study of identical twins

**DOI:** 10.3389/fpsyt.2026.1819962

**Published:** 2026-04-21

**Authors:** Sivert Drange, Jacob Cohen, Sys Stybe Johansen, Ben Dunkley, Mikael Palner

**Affiliations:** 1 Clinical Physiology and Nuclear Medicine, Department of Clinical Research, University of Southern Denmark, Odense, Denmark; 2 Department of Nuclear Medicine, Odense University Hospital, Odense, Denmark; 3 The Hospital for Sick Children, Toronto, ON, Canada; 4 Department of Pharmacology and Toxicology, University of Toronto, Toronto, ON, Canada; 5 Section of Forensic Chemistry, Department of Forensic Medicine, Faculty of Health and Medical Sciences, University of Copenhagen, Copenhagen, Denmark; 6 Neurobiology Research Unit, Copenhagen University Hospital, Copenhagen, Denmark

**Keywords:** case report, case study, cognitive flexibility, low doses, obsessive-compulsive disorder, psilocybin, twins

## Abstract

**Background:**

Obsessive-Compulsive Disorder (OCD) can present significant challenges to individuals mental health, characterized by intrusive thoughts and repetitive maladaptive behaviors. Recent research into alternative treatments has highlighted psychedelics, notably psilocybin, for their potential therapeutic benefits in various psychiatric disorders, including OCD. This case study evaluated the impact of self-administered, low-doses of psilocybin, commonly referred to as microdosing, on symptom reduction and cognitive flexibility in OCD, with a focus on identical twins discordant for the condition.

**Case presentation:**

The study documents the experiences of one twin diagnosed with OCD who began a regimen of low-doses of psilocybin containing mushrooms, while the other twin, unaffected by OCD, served as a comparison. Case X was diagnosed with OCD by a general practitioner in the Danish healthcare system. Following years of severe OCD, case X began a self-medicated regimen consisting of psilocybin containing mushrooms, corresponding to 1–5 mg of psilocybin, every 3^rd^ day. The other twin, case Y, who remained unaffected by OCD, and did not take psilocybin containing mushrooms. Cognitive flexibility was evaluated in both cases using a set-shift task. The affected twin reported a notable reduction in OCD symptoms, along with improvements in emotional regulation and overall well-being. However, despite these symptomatic improvements, deficits in cognitive flexibility remained present compared to the unaffected twin.

**Conclusion:**

This case study underscores the potential of low-doses of psilocybin as a promising avenue for mitigating symptoms of OCD. Nevertheless, the observed disparity in cognitive flexibility highlights the nuanced nature of OCD pathology, suggesting that while low-doses of psilocybin may alleviate certain symptoms, it may not fully address underlying cognitive impairments. Further research employing larger sample sizes and rigorous longitudinal designs is imperative to elucidate the mechanisms underlying the therapeutic effects of psilocybin low-doses in OCD, offering insights into its broader applicability as a treatment modality.

## 1 Introduction

Obsessive-compulsive disorder (OCD) is a disorder characterized by maladaptive patterns of repetitive thoughts and behaviors (1). The disorder is comprised of two main symptoms: obsessions and compulsions (1). Obsessions can be defined as unwanted thoughts, ideas, images, impulses and urges that are experienced as threatening, repulsive, or crude (2). Compulsions can be defined as repetitive behavior or mental acts that are conducted as a response to the distress caused by obsessions (2). The prognosis of OCD is often highly disabling, mainly affecting daily functioning and general well-being, which can also impose a heavy toll on the patients’ families (3). More specifically, OCD can lead to impairments in social functioning, general health, academic performance, and sleep (4, 5).

Anti-depressant drugs (selective serotonin inhibitors, SSRI’s) and behavioral therapy are the first lines of therapy for patients suffering from OCD; however, not all patients experience remission of symptoms following these treatments. It is estimated that between 40% to 60% of people afflicted by OCD continue to experience lingering symptoms even after undergoing appropriate treatment regimens of CBT sessions coupled with SSRI pharmacotherapy (6). The primary medical treatment for OCD today is SSRIs, which can cause adverse side effects, such as nausea, insomnia, sexual dysfunction and suicidal ideation (7). SSRIs do also not have an immediate effect; they can take weeks, or even months before attenuation of symptoms can be detected (8). Taking the limitations of today’s primary treatment into consideration and how burdening the disorder can be to the individual and the families, it is paramount to examine alternative treatment approaches to OCD.

An avenue of research that recently has recently received increasing attention relates to cognitive flexibility; the ability to change perspectives in response to alterations in environmental demands (9). Cognitive flexibility is a necessity for problem-solving, adapting to new situations, and learning from feedback (9). Several studies show that individuals with OCD have impairments in cognitive flexibility (10–12). Deficits in cognitive flexibility might therefore be an underlying mechanism contributing to the development and persistence of the disorder. It is therefore interesting to further investigate interventions targeted towards improving cognitive flexibility in OCD patients.

Psilocybin is a prodrug of psilocin, and a partial agonist to various receptors, with high affinity for the serotonin 5-HT_2_ family of receptors. Psilocybin is a novel promising drug for the treatment of OCD. Studies using psilocybin and other psychedelic drugs like LSD, have shown promise in enhancing cognitive flexibility and alleviating major depressive disorder, yet contradicting evidence exist Doss et al. (13) reported that a high dose (20-30mg/70kg) psilocybin increased cognitive flexibility in patients with treatment resistant depression, but the effect was not correlated to changes in functional connectivity as expected. In rats, acute psilocybin (1 mg/kg) increased while DOI, a more selective serotonergic psychedelic decreased cognitive flexibility in a set shift task (14), and initially decreased performance in a three-choice reversal learning task, while improving performance over time (15). This highlights a complex relationship between psilocybin and cognitive flexibility as also concluded by Meshkat et al. in a recent systematic review (16). In high doses, Psilocybin and LSD induce substantial hallucinogenic effects that can provoke anxiety, as such they require trained therapists and exceptional care when administrated. The acute hallucinations and anxiety may be limiting when used in individuals with mental health challenges. To address this, low-doses of psilocybin that do not produce any subjective psychedelic effects and show limited adverse effects (17), have become an anecdotal practice in people seeking to improve their mental health, performance and overall wellbeing (18–20).

At present, there is some evidence for psilocybin’s effects on symptoms of OCD, with a handful of clinical studies and case-reports (21–34). Of particular interest is the clinical trial by Moreno and colleagues (35) from 2006, which found that even low-doses (25-100µg/kg, 2-8mg in an 80 kg person) of psilocybin, originally intended as a placebo, also resulted in attenuation of OCD symptoms. Their recent follow-up study (33) confirmed the results in a from their first study, here repeated doses of 100 or 300 µg/kg showed reductions compared to active placebo. Pellegrini and colleagues (31) showed in a randomized clinical trial that a single dose of 10 mg psilocybin reduced primarily compulsions with less effect on obsessions. Furthermore, preclinical research show that repeated low-doses of psilocybin (0.05 mg/kg) can lower compulsive action and increase neuronal plasticity in wild-type rats (36), and that higher doses (4.4 mg/kg) lower compulsive actions in mice models of OCD (37, 38). Taken together, these studies indicate that psilocybin could contribute to symptomatic relief in OCD even at low-doses. Yet no studies to date have investigated the effect that low-doses of psilocybin may have on cognitive flexibility in this patient population.

Here we present an observational case-study in two identical twins on how low-doses of psilocybin affects symptoms of OCD and measurements of cognitive flexibility. We conducted a qualitative interview and performed an interdimensional – extradimensional set-shift task of reversal learning in each of the two identical twins, psilocybin was self-administrated and not provided by the researchers.

### 1.1 Case information

Case X is a male artist (painter) in his forties (40 years old at the time of interview) who has suffered from severe OCD most of his life. Case X was diagnosed with severe OCD (by a psychiatrist in the Danish healthcare system) in his early twenties, and later with a co-morbid Attention Deficit Disorder (ADD), while the nature of the OCD obsessions was not disclosed, the nature of his compulsions was of the pure-O type. The OCD diagnosis was confirmed by the study team doing interview using the ICD-10 guidelines. Despite numerous attempts at recovery, different therapies and medications, including SSRIs (brand and dose was not disclosed), nothing has been efficacious in reducing his symptoms of OCD. While the case received prescription of methylphenidate (Ritalin, below 80 mg as per described in the Danish treatment guidelines) that effectively reduced symptoms of ADD, his symptoms of OCD were unaffected. In 2020, case X started self-administering low-doses of psilocybin on a one-day on, two-days off schedule (the dose of active psilocybin was later confirmed to be between 1–5 mg per dose described below), in addition to prescribed methylphenidate and self-taught acceptance and commitment (ACT) based therapy and mindfulness therapy. Case X was in out-patient treatment, his primary practitioner did not participate in this report. Following this combination of medications for several months (as he recalls it took 6 months before the symptoms were reduced to a level noticeable by others), his OCD symptoms gradually attenuated, to the point where he now after 2 years described himself as free from OCD, while he continues to take psilocybin. However, as we did not know Case X before he started microdosing, these effects remain anecdotal. Before psilocybin, case X would be deemed treatment-resistant (39) (Treatment-resistant OCD can be defined as adequate trials of first-line therapies without achieving a satisfactory response). Today he describes himself as nearly symptom-free, as indicated by a score of less than 21 assed doing our interview, on the Obsessive Compulsive Inventory–Revised (OCI-R) (40), generally a score of above 40 indicates OCD. When asked about the causes of this dramatic improvement, case X attributes it to a combination of several factors. Firstly, he is adamant that the psilocybin has made it easier for him to withstand his obsessions and compulsions; that it has made them easier to manage. Case X does, however, emphasize that he believes that psilocybin on its own would not have reduced his OCD symptoms, but that it is efficacious in combination with his therapy. Case x did not report any adverse effects of psilocybin, despite the stigmatization “are you taking drugs” and the anxiety of obtaining psilocybin on the illegal marked.

Case Y is case X’s identical twin, a special needs teacher and, as expected with striking similarities to case X, considering both personality and behavior. Case Y has a wife and child whom he lives with and works within the health sector. Case Y and his twin brother grew up together and had a similar upbringing up until their early twenties. Case Y has also struggled with mental disorders, bipolar disorder, previously in his life, but not OCD and is currently considered healthy. When asked about how he would describe how low-dose psilocybin had affected his brother’s OCD symptoms, he responded in a way that closely mirrored his brother. He underlined that there were varied factors that contributed to his brother’s recovery, including psychotherapy and mindfulness therapy, in addition to psilocybin. Case Y expressed that he believes his brother’s improvement was largely due to the combination and synergistic effects of psilocybin and treatment. Case Y was impressed that his brother had shown significant relief of symptoms in a short period of time and now demonstrates a more optimistic perspective on his prognosis and his ability to cope with his OCD.

## 2 Methods and results

### 2.1 Psilocybin self-dosing and concentrations

Patient stated that he started using approximately 1 gram of hallucinogenic truffles sourced on the grey market then after a while switched to homegrown psilocybin mushrooms, which were grinded and dispersed in capsules containing approximately 0.1g each. Truffles are in general found to contain between 0.6 and 1.6 mg of psilocybin (41). A sample of the homegrown mushrooms were provided to the authors. Alkaloid content were extracted in methanol along with psilocin-d10, diluted with water, and analyzed using LC-MS as described previously (42). The home-grown mushrooms contained 3.9 mg psilocybin and 5.3 mg psilocin per capsule. 3 mg psilocybin are considered the threshold for perception when comparing the occupancy at 5-HT_2A_ receptors induced by psilocybin with the perceived psychedelic effect (42).

### 2.2 Intra-extra dimensional set shift task

The twins were administered the 3D Inter-extra dimensional set shift task (IED) and a qualitative interview. The IED is a neuropsychological test aimed at assessing cognitive flexibility, it has commonly been used to assess neurological and psychological disorders, including attention deficit disorders, autism spectrum disorder, and OCD. IED is a computerized adaption of the well-known Wisconsin Card Sorting Test (43), requiring participants to discriminate between stimuli and categorize them into sets. The objective of IED is to examine the participant’s ability to adapt their responses flexibly based on either punishing or rewarding feedback.

During the test, the participants are presented with several different stimuli, including different shapes, colors and number of figures. The stimuli are presented in two out of four different dimensions on the screen, and the participant must learn to categorize the stimuli based on a specific feature, as for example shape (1). The test moves through seven stages with stage 7 being the hardest. Case X, the twin with OCD, attempted to complete more trials (Case X: 48 vs Case Y: 19), but also made more errors (Case X: 25 vs Case Y: 8) in stage 7 of the test. This suggests more difficulty in completing stage 7 and thus impaired cognitive flexibility in the OCD affected twin.

## 3 Discussion

In this study, we have examined cognitive flexibility in two twin brothers, one twin self-medicated his OCD with low-doses of psilocybin and one twin was considered healthy, using a set shift learning task. The results showed that the healthy twin brother, case Y, needed fewer trials to complete the test and made fewer errors throughout the test. Although interesting, there are many confounding factors such as his brother’s diagnoses, medications and differing past histories that do not allow us to determine the leading factor.

Firstly, these results show that there are obvious differences in performance, even though the cases are twins and considered to perform quite similarly on such a test, barring any large differences in their environment or epigenetic changes (44). This is not the case, however. A possible explanation could be, as other studies (10–12) also have argued, that OCD causes impairments in cognitive flexibility, and that this is the reason for the differences in performance or that the practice of self-administration of low-doses psilocybin does itself induce deficits in cognitive flexibility.

Although most anecdotal reports indicate that low-doses of psilocybin can increase cognitive flexibility (45, 46), there are studies that suggest the opposite. A recent study by Cavanna and colleagues found in their study that the practice of low-doses psilocybin could cause impairments in cognitive functions, including negative effects on attention and decision-making (47). The study, however, was performed on healthy individuals with no evident impairments in cognitive flexibility. The study could, thus, not be representative of a population where cognitive flexibility is impaired, such OCD patients. Another study in rats, also showed that high doses of psilocybin impaired immediate cognitive flexibility in a three-choice reversal learning task (15). It can therefore be argued that the possible adverse effects psilocybin has on cognition do not adequately explain the differences in performance between the twins. Another study found that low-doses, improved creativity (48). Case X took up painting, which may be an indication of increased creativity in his case.

**Figure 1:**
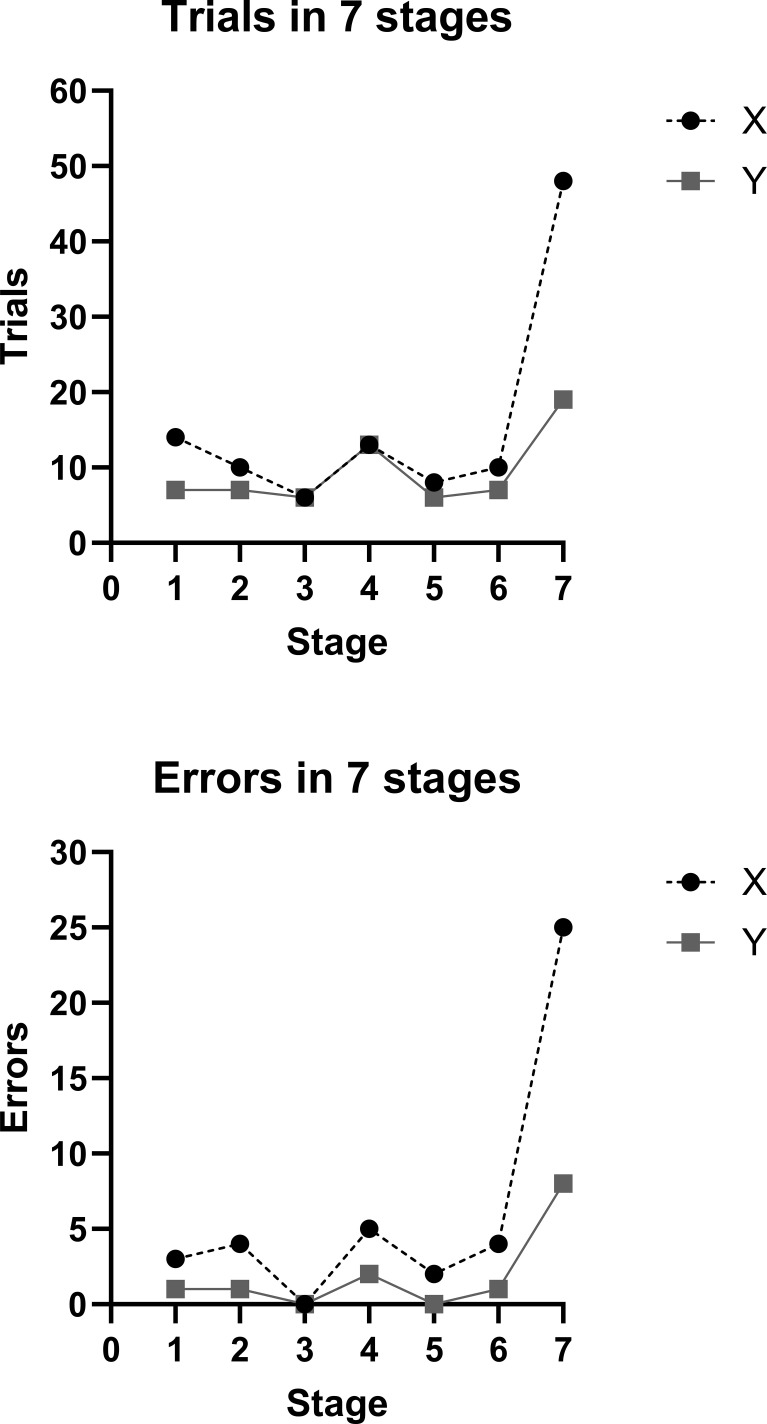
Total trials and errors made through the seven stages by either case X (OCD twin) or Y (healthy twin).

A possible explanation could be related to the performance on the extra-dimensional shift stage 7. Case X needed noticeably more trials to complete this stage and made more mistakes, compared to Case Y (**Figure 1**). At the extra-dimensional stage, the cases are required to make a substantial change in perspective, which relies on cognitive flexibility. Case X’s poor performance at this stage indicates that he may have gotten stuck in rigid thinking patterns and did not attempt novel problem-solving strategies. This could, imply that low-doses of psilocybin would not facilitate cognitive flexibility. In a recent study by Pacheco and colleagues they examined how the acute effects of psilocybin affect cognitive flexibility in rats (14). The results showed that psilocybin caused vast improvements in cognitive flexibility, but only when the rats were switching to behavioral strategies that had previously been learnt. The study by Pacheco and colleagues (14) does therefore imply that psilocybin does not facilitate novel problem-solving strategies, which are required at the extra-dimensional shift stage, but could make it easier to apply previously learnt strategies to novel situations. It is therefore possible that the facilitated utilization of previously learnt problem-solving strategies could have interfered with the case’s ability to attempt novel problem-solving strategies. Consequently, this would lead to poor performance on the extra-dimensional shift stage, as is the case with Case X. It is important to emphasize that this is speculative; nevertheless, it merits consideration as a promising pathway for future research.

This case study has several limitations that must be considered. First, the lack of a real-baseline assessment of Case X in both questionnaire and cognitive test is a limitation, while we did try to correct for this by included the comparison, his unaffected identical twin, Case Y. The self-administered nature of psilocybin low-doses introduces variability in dosing, adherence, and potential placebo effects, limiting the generalizability of the findings. It is almost impossible to know if the grey market products were of the same batch and same quality. We cannot quantify prior doses. Second, as a single-case design involving identical twins, the results may not be representative of the broader OCD population, requiring larger, more diverse samples to confirm these observations. Additionally, the study relies on qualitative self-reports and standard symptom assessments, which, while informative, lack objective biological markers or neuroimaging data to validate changes in brain function. The short-term nature of the study also prevents conclusions about long-term safety, efficacy, or potential adverse effects. Furthermore, the affected twin had a history of using other treatments, including methylphenidate and possibly other behavioral therapies, making it difficult to isolate the specific effects of low-doses of psilocybin and expectancy bias cannot be ruled out. Finally, while symptom relief was observed, persistent cognitive flexibility deficits might suggest that psilocybin low-doses may not fully address all aspects of OCD pathology, highlighting the need for further investigation into combination therapies or adjunctive interventions.

## 4 Case perspective

When discussing his compulsions after the low-doses regimen case X mentioned:

“*It will also be much easier, in my opinion, with psilocybin to learn to accept, it was difficult before. You can also learn to accept, and there are many who can say they can learn to accept, I could do that too, but you accept something that is very unpleasant still. With the psilocybin, you accept something that is very uncomfortable, and you are much freer. Before low-doses psilocybin, well it’s fine, I can accept and everything and it’s nice, it’s also liberating when you learn that technique, I’ve tried that, it was a relief but still a huge struggle*”.

“*Especially with regards to quality of life, because when you accept, you feel an extreme discomfort. With psilocybin this discomfort gets much smaller*”.

“*Psilocybin makes you feel your own self; you can move a lot more; you don’t do that with the SSRIs. You don’t get that perception that you feel yourself in a different way, it’s a completely different experience, it’s like you’re coming to yourself again, you’re gaining self-respect somehow, SSRIs cannot do tha*t”.

“*The experience of being 100% free of OCD occurs in periods, and the periods, over the last 3–4 months, increased from 1 to 2 days, they get longer and longer. It’s a small miracle*”.

### 4.1 Implications of findings

A crucial question that remains is how the results from the IED can be related to the attenuation of OCD symptoms experienced by Case X. Essentially, the results indicate that while low-doses of psilocybin reduced perceived symptoms they did not improve performance in the extra-dimensional set shifts. As such low-doses of psilocybin may not increase cognitive flexibility and may not target the full symptomatology of OCD, as also described be Pellegrin et al. in their recent clinical trial with a single 10 mg dose (31). An individual with OCD may be accustomed to alleviating distressing feelings by engaging in compulsive behaviors, but after psilocybin, may attempt to accept the distressing feeling rather than suppress them be compulsive behaviors. This would be an extra-dimensional shift, and an attempt at novel problem-solving strategies. If low-doses of psilocybin, in fact, make it harder to perform such shifts, it would not be advantageous to OCD patients, and could make it more difficult to break out of repetitive patterns. This is, however, quite the contradiction to case X’s experience with psilocybin, who is adamant that it has helped him. A possible explanation could be related to Pacheco and colleagues’ (2023) findings: That psilocybin makes it easier to apply previously learnt problem-solving strategies to novel situations. As mentioned, Case X took part in mindfulness and acceptance therapy simultaneously with the low-doses practice, where he learned new ways to manage his OCD. It is possible that psilocybin made it easier for him to apply these techniques to novel situations in his everyday life, consequently helping him break out of the repetitive OCD cycle.

## 5 Conclusion

An avenue of research that has gained a lot of traction in recent years is the use of psychedelics in the treatment of mental disorders, including psilocybin. Through both interviews and a reversal learning test, we aimed to examine the effects that low-doses of psilocybin have on cognitive flexibility. It is. however, important to clarify that the object of this study was not to establish any causality, only to gather some preliminary data on a subject that lacks comprehensive understanding. The results indicated that low-doses of psilocybin did not amend the cognitive flexibility deficits in the case with OCD yet still made it easier to overcome OCD symptoms and increase his quality of life. A potential role for low-doses of psilocybin could be to facilitate the use of techniques learnt in psychological therapy making them more efficacious. Albeit speculative, it merits consideration as a potential pathway for future research to investigate further. Future investigations into the therapeutic potential of low-dose psilocybin for OCD should prioritize methodological sound randomized, placebo-controlled trials. Neuroimaging techniques, such as fMRI and EEG or MEG, will be essential for elucidating the mechanisms of action within OCD-related neural circuits. Comparative effectiveness studies against established treatments, including SSRIs and CBT, are necessary to determine if psilocybin can be a replacement or adjunct with current therapies. Longitudinal studies are required to comprehensively evaluate the safety profile, sustained efficacy, and long-term impact of psilocybin treatment. Furthermore, given the observed persistence of cognitive flexibility deficits despite symptom reduction, research should explore adjunctive therapies to address the cognitive effects.

## Data Availability

The raw data supporting the conclusions of this article will be made available by the authors, without undue reservation.
